# Thanks to Reviewers 2017

**DOI:** 10.5334/pb.427

**Published:** 2017-12-12

**Authors:** Editorial Team

**Affiliations:** Psychologica Belgica, BE

## Abstract

All manuscripts published in *Psychologica Belgica* have been assessed conscientiously and unselfishly by expert reviewers. The quality of our journal totally depends on their valuable and constructive criticisms to the authors. Both the editors and the authors highly appreciate the input and dedication of all our reviewers. Many thanks.

**Cristina Antunes**, University of Trás-os-Montes and Alto Douro, Portugal

**Laurent Bègue**, Université de Grenoble, France

**Neda Bebiroglu**, Université catholique de Louvain, Belgium

**Katrijn Brenning**, Ghent University, Belgium

**Emilie A. Caspar**, Université libre de Bruxelles, Belgium

**Liesje De Backer**, Ghent University, Belgium

**Elien De Caluwé**, Ghent University, Belgium

**Egon Dejonckheere**, Katholieke Universiteit Leuven, Belgium

**Jennifer Denis**, Université de Mons, Belgium

**Carlo Garofalo**, Tilburg University, Netherlands

**Yannick Griep**, University of Calgary, Canada

**Fransciska Krings**, Université de Lausanne, Switzerland

**Frank Laroi**, University of Bergen, Norway

**Baptist Liefooghe**, Ghent University, Belgium

**Koen Luyckx**, Katholieke Universiteit Leuven, Belgium

**David Magis**, Université de Liège, Belgium

**Igor Marchetti**, Ghent University, Belgium

**Gaetan Mertens**, Ghent University, Belgium

**Christian Monseur**, Université de Liège, Belgium

**Carmen Moret-Tatay**, Universidad Católica de Valencia, San Vicente Mártir, Spain

**Arjen Noordhof**, University of Amsterdam, Netherlands

**Emma Onraet**, Ghent University, Belgium

**Michael Rönnlund**, Umeå University, Sweden

**Gina Rossi**, Vrije Universiteit Brussel, Belgium

**Laurence Rouselle**, Université de Liège, Belgium

**Jeroen Stouten**, KU Leuven, Belgium

**Jolene van der Kaap-Deeder**, Ghent University, Belgium

**Anja van der Voort**, Leiden University, Netherlands

**Marie Vander Haegen**, Université de Liège, Belgium

**Maarten Vansteenkiste**, Ghent University, Belgium

**Tim Vantilborgh**, Vrije Universiteit Brussel, Belgium

**Evy Woumans**, Ghent University, Belgium

**Sofie Wouters**, KU Leuven, Belgium

**Jia Yueming**, ABA Research & Educational Consulting, USA

